# Batch and Flow Synthesis of CeO_2_ Nanomaterials Using Solid-State Microwave Generators

**DOI:** 10.3390/molecules27092712

**Published:** 2022-04-22

**Authors:** Cristina Rodríguez-Carrillo, Juan Torres García, Miriam Benítez, Jamal El Haskouri, Pedro Amorós, Jose V. Ros-Lis

**Affiliations:** 1 Departamento de Química Inorgánica, Universitat de València, Doctor Moliner 50, P.O. Box 73, 46100 Butjassot, Spain; cristina.rodriguez-carrillo@uv.es (C.R.-C.); juan.torres-garcia@uv.es (J.T.G.); miriam.benitez@uv.es (M.B.); 2 Institut de Ciència dels Materials, Universitat de València, P.O. Box 22085, 46071 Valencia, Spain; jamal.haskouri@uv.es (J.E.H.); pedro.amoros@uv.es (P.A.)

**Keywords:** CeO_2_, nanoparticle, microwave-assisted synthesis, solid-state generator, flow chemistry

## Abstract

Microwave-assisted synthesis in combination with flow synthesis offers an interesting approach to develop faster and more sustainable procedures for the preparation of homogeneous nanomaterials. Recently, solid-state generators of microwaves appeared as a tool with improved control over power and frequency. Cerium oxide, despite its excellent catalytic activity, has not been prepared before using solid-state generators or microwave-assisted flow chemistry. We report a procedure for the preparation of nanoparticulated CeO_2_ (around 4 nm) under 2.45 GHz microwaves in only 30 s. The materials are further calcined at 800 °C to increase particle size, with a better defined particle size and crystallinity. The procedure was tested in batch at pH 11 and 12 and diverse potencies, and the products were characterized by TEM, XRD, DLS, and N_2_ adsorption–desorption isotherms. The materials were similar at the diverse pH values and potencies. XRD confirms the crystallinity of the CeO_2_ material with a fluorite-like structure. They are composed of particles around 40 nm that aggregate as structures of around 100 nm. The procedure was successfully adapted to flow synthesis, obtaining materials with structure and properties equivalent to batch synthesis. The batch and flow materials offer peroxidase properties, opening the door for their use as ROS scavengers.

## 1. Introduction

Nanomaterials have attracted broad attention in recent decades due to their high surface/volume ratio and novel properties derived from nanoscale. Among them, nanoscopic cerium oxide offers interesting catalytic properties due to the unique electronic configuration of the lanthanide atom, the reduction potentials, the reversible conversion between the oxidation states of Ce^3+^ and Ce^4+^, and its oxygen buffering capacity. The close thermodynamic stability of CeO_2_ and Ce_2_O_3_ favors an easy and reversible transition between these two compounds, giving rise to a range of partially reduced CeO_2−x_ phases that serve as oxygen reservoirs by creating or eliminating oxygen vacancies. The use of ceria has been explored in hydrogen production [1], as an oxygen capacitator in automotive three-way catalysts [2], as a chemosensor and photocatalyst [3,4,5], and in ion conducting membranes [6]. 

Reactive oxygen species (ROS) include compounds such as singlet oxygen, superoxide, hydrogen peroxide, and hydroxyl radical. Although these substances are naturally produced in cells as a consequence of oxidation metabolism in the mitochondria, they are highly reactive and potentially harmful to living organisms. An excess of ROS induces oxidative stress, damages biomolecules such as proteins, lipids, and nucleic acids, and finally induces apoptosis. Thus, living organisms limit the concentration of ROS at a cellular level by producing enzymes (for example, superoxide dismutase, glutathione peroxidase or catalase) or antioxidants [7]. Recently, in biological applications, CeOx was explored as a scavenger of reactive oxygen species, mimicking diverse enzymatic reactions [8]. 

Microwaves (MWs) are a source of electromagnetic radiation lying between infrared and radiofrequencies that have shown interesting properties in chemical reactions. In comparison with conventional synthesis, MWs offer a higher synthesis rate and shorter reaction times (typically reduces the reaction time from hours to minutes), more homogeneous products, smaller particle size, narrower particle size distribution, higher purity, higher yields due to minimization of side products, selective heating, lower power consumption (environmentally friendly), etc. [9] Considering these advantages, it is not surprising that microwaves have been used previously for the synthesis of cerium oxide. Depending on the reagents and the synthesis conditions, the size and shape of the materials vary. They have been prepared as aggregates of particles in the nanometric to micrometric range in less than 1 h of irradiation [10,11]. They could be prepared even as 2 nm particles in the presence of PEG in only 10 min [12]. Other shapes include nanorods/nanowires [13,14] or microplates through the calcination of Ce(OH)CO_3_ [15]. Further advances in the microwave-assisted synthesis of ceria include the preparation of a mesoporous material departing from a CMK-3 carbon structure as a template [16] or the preparation of mixed oxides such as Ce_x_Sm_1−x_O_2_ [17]. In all cases, the materials were prepared in batch using magnetrons as a source of microwave energy.

Despite all the advantages mentioned in the previous paragraph, conventional microwave-assisted procedures of synthesis based on the use of magnetrons as an energy source offer some restrictions. The combination of solid-state microwave generators with flow synthesis offers an interesting approach to overcome such restrictions. Solid-state generators offer greater simplicity in the implementation and power management, with a longer duration of the power source in comparison to magnetrons [18]. On the other hand, one of the main limiting factors of microwave application is the small amount of material obtained in each batch due to the MW properties and the design of the reactors. The use of flow synthesis strategies can allow achieving large amounts of product as the materials are produced continuously [19]. So far, neither of the two strategies (the use of solid-state generators or flow synthesis) has been applied for the preparation of nanomaterials based on cerium oxide. In this work, the possibilities offered by batch and flow synthesis using solid-state microwave generators for the preparation of nanoscale cerium oxide were evaluated. Considering the possibility of using CeO_2_ as a nanozyme with ROS-scavenging properties, the peroxidase activity was evaluated and verified.

## 2. Results and Discussion

### 2.1. Synthesis Procedure

**Scheme 1:**
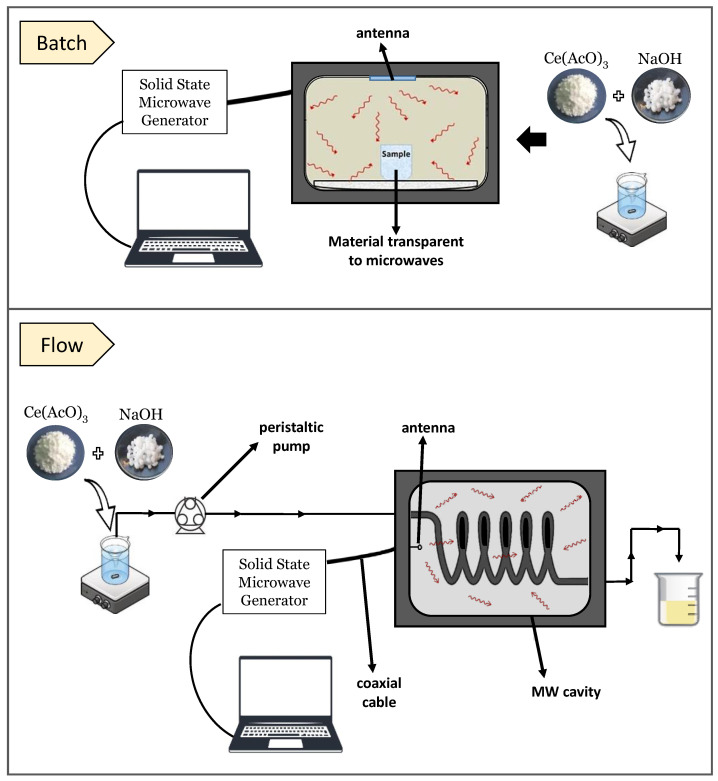
Scheme describing the setup for the synthesis in batch and flow.

The synthesis was carried out using static (batch) and dynamic (flow) reactors powered by microwave solid-state generators (see Scheme 1). In batch, diverse values of pH, power, and irradiation time were tested (see Table 1). The notation used for the materials was B or F depending on whether the synthesis was in batch or flux, 11 or 12 as a function of the pH, and L, M, or H for low (50 W), medium (100 W), or high (200 W) power. NC was added before the name for the non-calcined materials.

The solid-state generator allowed us to tune finely the power. In all cases, the temperature of the solution reached temperatures around 90 °C. As expected, when the power is increased, the time necessary to reach this temperature is reduced. To our knowledge, 30 s is the shortest time reported for CeO_2_ synthesis in solution [10,11,12,13,14,15]. Considering the nominal power and irradiation time and the temperature reached by the solution, approximately 50% of the energy delivered by the solid-state source is transformed into heat in the solution. Part of the energy returns to the solid-state microwave generator and is dissipated as heat in the energy source. The percentage increases when the time is reduced in agreement with a lower heat transfer to the reaction flask and the preferential heating of the water in comparison with glass under microwave irradiation [20]. In agreement with the resemblance in the composition, the dielectric properties of the solutions and, therefore, the absorption of microwaves were similar at pH 11 and 12.

Furthermore, a non-calcined material prepared at pH 12 with irradiation at 200 W for 30 s was dried and collected to evaluate the effect of the calcination in the structure and morphology of the cerium oxide (NCB12H). Regarding flow synthesis, in agreement with the similarity of the materials obtained in batch at diverse synthesis conditions (see below), the fastest synthesis at pH 12 was selected. The properties of the material prepared in batch and flow synthesis were characterized by X-ray diffraction (XRD), transmission electron microscopy (TEM), dynamic light scattering (DLS), and N_2_ adsorption–desorption isotherms. Additionally, the as-made materials were characterized to evaluate the effect of calcination at 800 °C.

### 2.2. Crystallinity of the Materials

**Figure 1:**
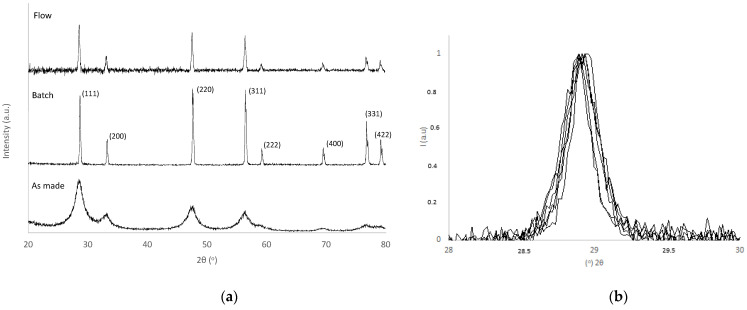
XRD studies. (**a**) XRD of B11M, with the hkl index of each plane indicated between brackets; (**b**) detailed XRD 111 peak of the six batch materials (BXXX) prepared under batch conditions, with the peaks normalized for comparison purposes.

XRD was used to determine the chemical structure of the materials obtained during the synthesis. As can be seen in Figure 1a, the peak assignment of the B11M diffractogram corresponded to typical CeO_2_ in a cubic fluorite-like structure. The most intense peaks at 2θ values of 28.7°, 47.6°, and 56.5° could be assigned to the (111), (220), and (311) hkl planes, respectively, with a cell parameter of a = 5.403 Å. No peak corresponding to Ce_2_O_3_ was found. The XRD of the remaining materials showed peaks in the same position and with similar full width at half maximum (FWHM) (Figure 1b), indicating that, under our experimental conditions, the phase formed and crystallinity of the materials were similar.

The yellow material obtained directly from the microwave NCB12H (dried but not treated at 800 °C) was also characterized, revealing the peaks characteristic of CeO_2_ (Figure 1a). This result suggests that a fast treatment in the microwave is enough to obtain Ce(IV) oxide material with a crystalline structure. However, the full width at half maximum was significantly higher than in the case of the calcined materials, suggesting a lower crystallinity or lower particle size. In the case of the material prepared under flow conditions, XRD peaks were in the same positions with the same relative intensity as the materials prepared in batch. This result confirms that flow synthesis can be applied for the preparation of CeO_2_ nanomaterials.

### 2.3. Particle Size and Morphology

**Figure 2:**
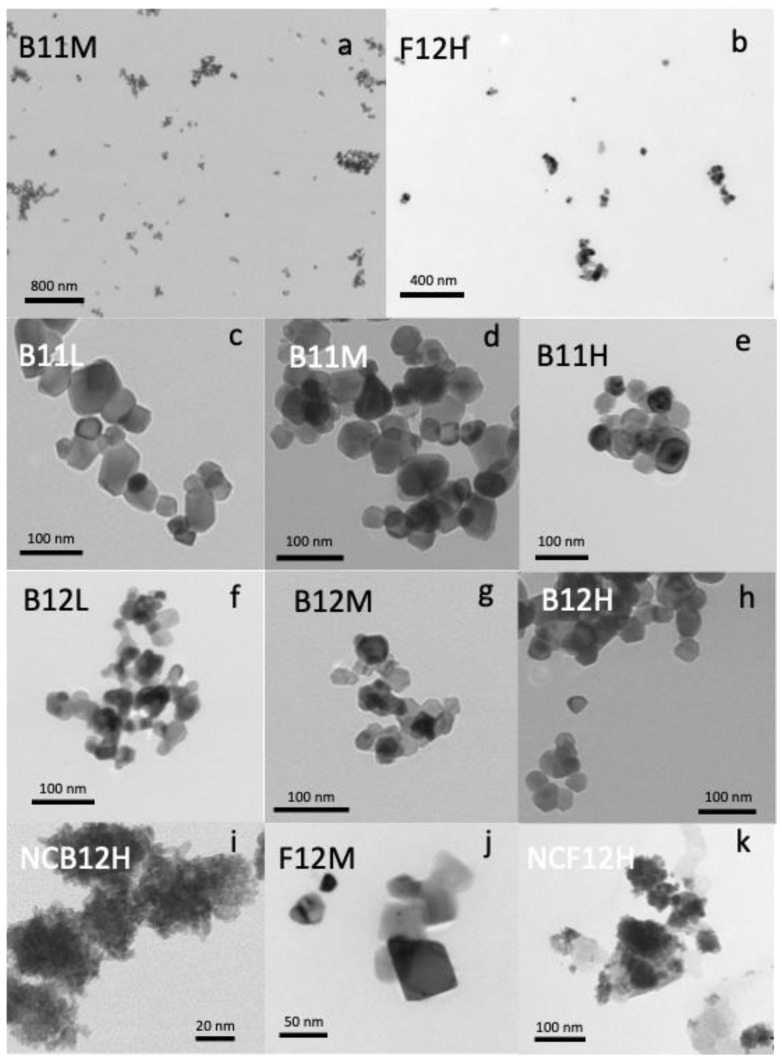
TEM images of the CeO_2_ materials: (**a**) general view of B11M, (**b**) F12H, (**c**) B11L, (**d**) B11M, (**e**) B11H, (**f**) B12L, (**g**) B12M, (**h**) B12H, (**i**) NCB12H, (**j**) F12H, and (**k**) NCF12H. B (batch); F (flow); 11 or 12 indicates the pH; L, M, or H for low (50 W), medium (100 W), or high (200 W) power. NC is added before the name for the non-calcined materials.

TEM analysis was used to determine the morphology and organization of the particles. As can be seen in Figure 2, in the batch (Figure 2a) and flow synthesis (Figure 2b) regimes, we obtained individual particles together with aggregates, even after applying ultrasound to disaggregate the material. It seems that the particles tended to aggregate during the synthesis in solution and filtration, and the aggregates were consolidated during calcination at high temperature (800 °C) over several hours. At higher magnification, TEM images illustrate that the materials prepared under batch conditions BXXX (Figure 2c–h) were similar. They were composed of nanometric CeO_2_ particles with irregular shapes (from spherical to cubic). Measurements of particle size show that the materials were composed of particles around 40 nm, and a distribution in the range of 20 to 60 nm could be found (see Table 2). The shape and size of the particles were similar to the values reported in microwave-assisted synthesis using magnetrons [10,11]. To evaluate if the variation in the synthesis conditions was able to induce significant differences in the particle size, the particle size distribution was analyzed with statistical tools. The particle distribution did not follow a normal distribution; thus, the Kruskal–Wallis test was applied. In this case, the main differences were observed upon variation of the pH. At a lower pH (11), the particles were statistically bigger than at pH 12; thus, a growth of the particles was observed. Power offered only a minor effect; however, at 50 W and pH 12, it was observed that the particles were smaller in comparison with the irradiation at higher power. In studying the size and morphology of the material prepared under flow synthesis in comparison with batch conditions, TEM imaging showed a morphology and size similar to the materials prepared in batch (Figure 2j). Data analysis of the particle size distribution revealed that the material was assigned to the same group as the batch materials prepared at pH 12; thus, we were able to reproduce batch synthesis under flow conditions.

Regarding the effect of the calcination step on the morphology of the material, by contrast to the calcined materials that offered defined dense particles, the material obtained directly from the microwave reactor was mainly composed of smaller nanoparticles (ca. 4 nm) (Figure 2i,k). These primary nanoparticles were consolidated during the treatment at high temperature to form the final particles. In agreement with the data obtained from XRD, the non-calcined material was crystalline in nature. Since a reduced crystal size could explain the wide XRD signal observed, the Scherrer equation was applied to evaluate the crystallite size from the full width at half maximum of the XRD peaks. A crystallite size of 4.9 was calculated. Although a particle can be formed by several crystals, in our case, the fact that the size of the crystallites determined from the XRD data and the application of the Scherrer equation was similar to the size of the nanoparticles observed by TEM suggests that, under our preparative conditions, a very fast nucleation step occurred, generating crystalline ceria from the beginning of the process. As expected, under relatively homogeneous conditions with rapid heating under microwave, the initial steps in the formation of materials from molecular sources consisted of the formation of small primary nanoparticles (ca. 4 nm and even some smaller), which grew by aggregation/coalescence and evolved to larger crystalline nanoparticles when subsequent calcination treatments were applied. 

The textural porosity formed by the aggregation of the little particles was also confirmed by N_2_ gas adsorption (see Supplementary Figure S1). In the case of the material NCB12H, an area of 144 m^2^·g^−1^ was found, in agreement with the presence of a textural porosity due to the 4 nm particles observed in TEM. The N_2_ adsorption–desorption isotherm of the NCB12H sample showed a gradual nitrogen adsorption over the entire pressure range. The application of the Barrett–Joyner–Halenda model (BJH) model allowed determining a pore volume of 0.1 cm^3^·g^−1^. However, there were no well-defined pores. As mentioned before, the porosity (irregular) at both the micro- and the mesoscopic scales of a textural type was related to the voids between the primary nanoparticles. The nanoparticle aggregates observed through TEM for the non-calcined sample usually generated some cage-like pores that were responsible for the hysteresis loop observed in the isotherm. On the contrary, when the material was calcined, the specific surface was drastically reduced to 0.11 m^2^·g^−1^, confirming the complete loss of textural-type porosity. Thus, in the calcined material, the low measured area was due solely to the outer surface of isolated or poorly aggregated crystalline nanoparticles.

**Figure 3:**
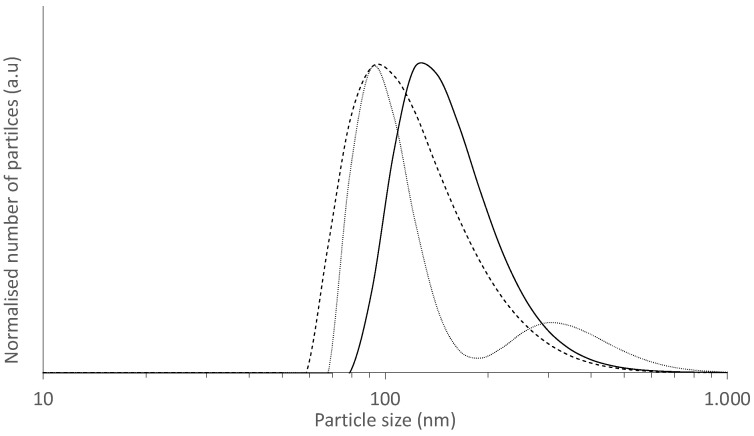
Dynamic light scattering spectra: B11M, straight line; B12M, dashed line; F12H, dotted line.

The particle size was also determined through DLS. This technique measures a wide number of particles and informs us about the degree of aggregation and the size of the aggregates in solution. As can be seen in Figure 3, B11M exhibited a peak in the number of particles around 122 nm (other peaks could be calculated in the deconvolution at 173 nm), while B12M exhibited a peak at 91 nm (with a tail calculated by deconvolution at 137 nm). Conversely, F12H offered two peaks at 91 nm and 314 nm. In agreement with TEM, at pH 12, the particles offered a lower size than at pH 11, and the change in the particle size at both pH values measured with DLS was proportional to the values measured with TEM. In the case of F12H, in addition to the peak at 91 nm found for the batch synthesis, a peak at larger size (314 nm) was also identified, indicating a higher aggregation possibly due to synthesis conditions in the flow reactor. DLS measured a greater hydrodynamic radius than measured with TEM; however, in this case, the wide difference suggests that the material could have been formed by a relevant number of little aggregates conformed by few particles. In any case, the resulting material remained in the nanometric range, well below 1 µm. 

### 2.4. ROS Scavenging Properties

**Figure 4:**
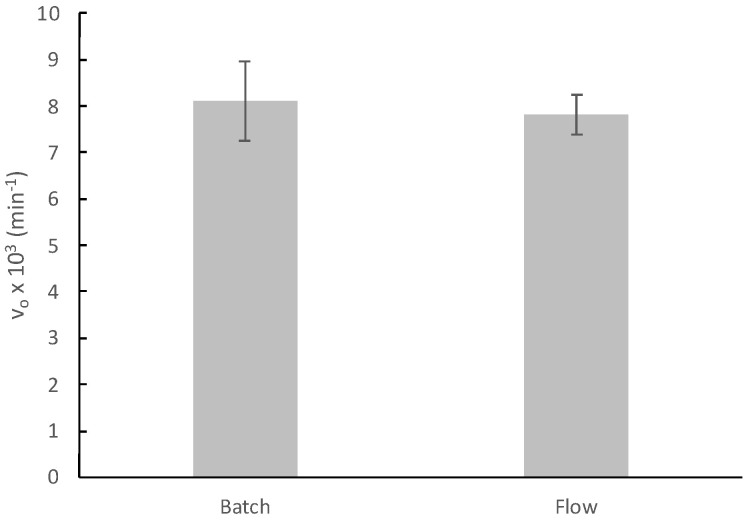
Peroxidase activity of the CeO_2_ materials.

As noted above, cerium oxide offers interesting redox properties that have been used for catalytic application. In our case, we were interested in the ROS-scavenging properties with future biological applications in mind. We studied the peroxidase activity of the materials prepared under batch and flow conditions (Figure 4). The peroxidase-like activities of the materials were studied by the catalytic oxidation tests toward 3,3′,5,5′-tetramethylbenzidine (TMB) with the assistance of H_2_O_2_. Upon oxidation, TMB develops a blue color; thus, higher absorbances correspond to higher peroxidase activity. In our case, we found a reaction rate of approximately 8 × 10^−3^ min^−1^. The equivalent activity found for the materials prepared under batch and flow conditions was in agreement with the similarities found for both type of materials during the characterization, confirming that our flow synthesis strategy allowed us to obtain cerium oxide materials with properties similar to batch synthesis.

### 2.5. Energy Efficiency

Currently, there is a strong tendency toward the development of synthesis processes that are more sustainable and have a lower carbon footprint. One of the advantages of microwaves is the significant reduction in the reaction time. In comparison with a conventional microwave reactor, solid-state microwave generators allow the modulation of power and frequency. A low reaction time, together with the use of low potencies, affords a synthesis process with very low energy consumption. In our case, the reaction time was only 30 s. This procedure is much faster than other microwave-assisted processes previously reported in the literature and conventional methods. The energy consumed in our case was 0.010 kWh (7 g equivalent of CO_2_) per synthesis and 0.14 W·h·mg^−1^ (98 mg equivalent of CO_2_ per mg of CeO_2_). This amount is almost two orders of magnitude lower than the 0.55 kWh (392 g equivalent of CO_2_) used by an 800 W magnetron in 30 min, conditions found in previously reported procedures [11]. 

## 3. Materials and Methods

### 3.1. Chemicals

Cerium(III) acetate hydrate (Ce(CH_3_CO_2_)_3_·xH_2_O), 99% metal basis (Sigma-Aldrich, St. Louis, MO, USA), sodium hydroxide (NaOH) >98% (Sigma-Aldrich), and hydrochloric acid solution 1 N (Scharlau, Barcelona, Spain) were used to synthesize the nanoparticles of this work. Water used in this investigation was deionized. Hydrogen peroxide (H_2_O_2_) 35% (Panreac) and 3,3′,5,5′-tetramethylbenzidine (TMB) (Sigma-Aldrich, St. Louis, MO, USA) were used to determine peroxidase activity.

### 3.2. Synthesis of the CeO_2_ Nanomaterials

The reaction was performed in a microwave reactor purchased from Microbiotech (Valencia, Spain). It consists of a microwave chamber equipped with a 200 W solid-state generator at 2.45 GHz. For batch synthesis, 0.95 g of cerium acetate and 0.48 g of sodium hydroxide (1:4 molar ratio) were dissolved in 50 mL of deionized water, and the pH was adjusted to 11 or 12, depending on the material. Right after, 10 mL was transferred to a microwave glass vial that was placed in the microwave oven with the power set at 50, 100, or 200 W. Irradiation times were established at 0.5, 1.5, or 2.5 min for 200, 100, or 50 W, respectively. The resulting yellow precipitate was washed with water and ethanol to neutral pH. Drying and calcination were performed in a conventional furnace. For drying, the oven was preheated at 80 °C before its use and maintained overnight. The furnace for calcination was set with a heating ramp at 20 °C·min^−1^ up to 800 °C. The temperature was maintained for 6 h and allowed to cool down until room temperature. The samples remained inside the furnace throughout the calcination process.

In flow synthesis, 4 g of Ce(AcO)_3_ and 2 g of NaOH were dissolved in 200 mL of de-ionized water with a pH of 12. The mixture was pumped into the microwave oven, so that the sample was irradiated for 30 s at 200W. The resulting yellow precipitate was washed with water and ethanol to neutral pH. Lastly, the solid was dried overnight at 80 °C and calcined at 800 °C for 6 h.

### 3.3. Characterization of the Materials

The powder X-ray diffraction pattern of the nanoparticles was obtained using a powder X-ray Diffractometer (Avance A25, Bruker Cooperation, Billerica, MA, USA). The sample was scanned over the required range for 2θ values (10–80°). The size and shape of nanoparticles was obtained by transmission electron microscopy (TEM) using a HITACHI HT7800 120 KV. The particle size analysis for the sample was carried out using the particle size analyzer Zetasizer nano series of Malvern Instruments. Nitrogen adsorption–desorption isotherms were recorded in an automated Micromeritics ASAP2010 instrument. Prior to the adsorption measurements, the samples were outgassed in situ in vacuum (10^−6^ Torr) at 110 °C for 15 h to remove adsorbed gases. The specific surface area was determined by applying the Brunauer–Emmett–Teller (BET model) from the adsorption data within the low-pressure range. Pore volume was calculated following the Barrett–Joyner–Halenda model (BJH). The UV/visible spectrum for peroxidase activity was obtained using a Jasco V-770 spectrophotometer.

### 3.4. Determination of the Peroxidase Activity

The peroxidase activity was measured by mixing the material, H_2_O_2_, and TMB, and the change in color was determined in the UV/visible absorption range following Cao’s protocol [21]. Briefly, 70 μL of 1.0 mg·mL^−1^ CeO_2_ material was added to 400 μL of 50.0 mM phosphate buffer (pH = 4.0) at room temperature, followed by the addition of 100 μL of 8.0 mM TMB, 100 μL of 25.0 mM H_2_O_2_, and 330 μL of Milli-Q water. Subsequently, the reaction solutions were incubated at room temperature for 7 min. Afterward, the UV/visible absorption at 652 nm was measured using a Jasco V-770 spectrophotometer. 

### 3.5. Data Analysis

The Scherrer equation was used for the calculation of the XRD crystals. It relates the size of crystallites to the broadening of a peak in a diffraction pattern using K = 0.9 [22]. Statistical analysis was performed using the SPSS software. The comparison of the particle size was performed using the independent-sample Kruskal–Wallis test at a significance level of 0.05. The significance values were adjusted by Bonferroni correction for multiple tests. The conversion of kWh to g of CO_2_ was calculated using the Avoided Emissions and Generation Tool (AVERT) of the Environmental Protection Agency (EPA) [23]. 

## 4. Conclusions

We validated the viability of using a solid-state microwave generator for the synthesis of cerium oxide. This technology can be applied in batch or in flow synthesis. The flow microwave-assisted synthesis of CeO_2_ has not been previously reported. The materials prepared under microwave irradiation showed a typical fluorite-like structure and were formed by 4 nm particles. After calcination at 800 °C, the particles grew to the 30–45 nm range with better defined shape and improved crystallinity, which aggregated in conglomerates of 80–300 nm. Variations in the power/time did not significantly affect the size and structure of the materials. Flow synthesis was able to reproduce the particle size, morphology, crystallinity, and peroxidase activity of the materials prepared using the batch synthesis procedure. By contrast, when the pH was reduced from 12 to 11, a slight growth in the particle size was observed. The materials offered equal peroxidase activity when prepared under batch and flow conditions. These results open the door toward a wider use of solid-state generators in the synthesis of materials and confirm their potential of preparing ceria.

## Figures and Tables

**Table 1:** Summary of synthesis conditions.

Material	Reactor	pH	Power (W)	Time (min)
B11L	Batch	11	50	2:30
B11M	Batch	11	100	1:00
B11H	Batch	11	200	0:30
B12L	Batch	12	50	2:30
B12M	Batch	12	100	1:00
B12H	Batch	12	200	0:30
NCB12H ^1^ F12H	Batch Flow	12 12	200 200	0:30 0:30
NCF12H ^1^	Flow	12	200	0:30

^1^ Non-calcined materials.

**Table 2:** Measurement of particle size using TEM photographs.

Material ^1^	n^2^	Particle Size $\left(\bar{x}\;\pm \;SD\;\mathrm{nm}\right)$	Particle Size Median (nm)
B11L ^a^	146	45 ± 14	44.3
B11M ^a^	158	43 ± 15	42.0
B11H ^a^	78	46 ± 15	43.6
B12L ^b^	117	30 ± 10	28.4
B12M ^c^	107	40 ± 20	33.3
B12H ^c^	159	37 ± 11	35.8
F12H ^b,c^	80	34 ± 15	32.3

^1^ Formation of groups using the Kruskal–Wallis test (*p* = 0.05), where the same letter indicates the same group. ^2^ Number of particles measured.

## Data Availability

Not applicable.

## Supplementary Materials

The following supporting information can be downloaded at: https://www.mdpi.com/article/10.3390/molecules27092712/s1, Figure S1: N2 adsorption-desorption isotherms of the material NCB12H.
