# Supplementary material for: Batch and Flow Synthesis of CeO2 Nanomaterials Using Solid-State Microwave Generators
Source: Molecules. 2022 Apr 22;27(9):2712. doi: 10.3390/molecules27092712 (PMC9101767; doi:10.3390/molecules27092712)
Supplement: Supplementary file 1 [file molecules-27-02712-s001.zip › molecules-1645823-supplementary.pdf]

ELECTRONIC SUPPLEMENTARY MATERIAL

BATCH AND FLOW SYNTHESIS OF CeO_2 NANOMATERIALS USING SOLID STATE MICROWAVE GENERATORS.

Cristina Rodríguez-Carrillo, Juan Torres García, Miriam Benítez, Jamal El Haskouri, Pedro Amorós and Jose V. Ros-Lis

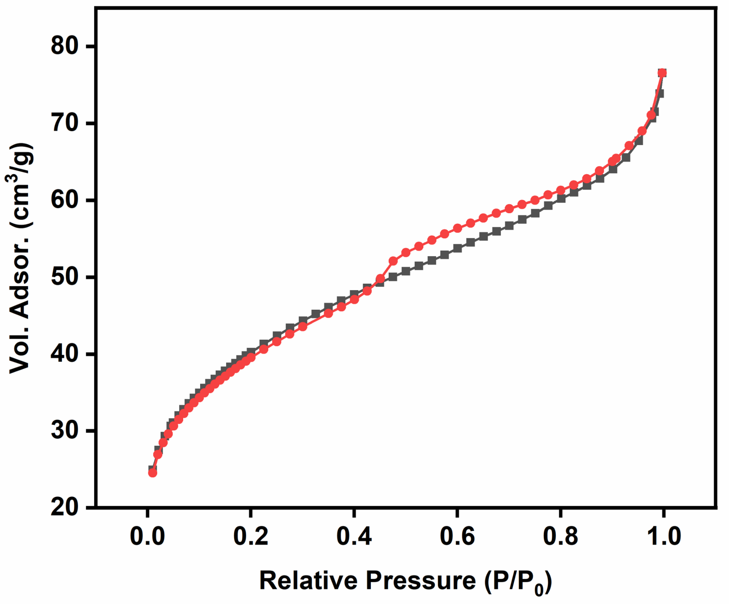

Figure S1: N_2 adsorption-desorption isotherms of the material NCB12H.
